# Digital literacy, phubbing, and mental well-being in the digital age: a study on young university athletes

**DOI:** 10.3389/fpsyt.2025.1638959

**Published:** 2025-08-12

**Authors:** Tolga Tek, Arif Özsari

**Affiliations:** ^1^ Faculty of Sport Sciences, Selcuk University, Konya, Türkiye; ^2^ Faculty of Sport Sciences, Mersin University, Mersin, Türkiye

**Keywords:** digital literacy, phubbing, mental well-being, young university athletes, sport

## Abstract

Digital literacy has now emerged as a pivotal determinant of individuals’ social, psychological, and mental responses in contemporary society. This study examined the relationships between digital literacy, phubbing behaviors, and mental well-being among young university athletes. The sample consisted of 224 students (mean age = 20.91; SD = 1.98) from the sports sciences faculty of a state university, including 109 females (48.7%) and 115 males (51.3%), who participated voluntarily. Three validated measurement instruments were employed, with confirmatory factor analysis conducted to establish scale reliability and validity. Statistical analyses included descriptive statistics, correlation analysis, and regression analysis to explore variable relationships within the research model. Correlation analysis detected a significant positive moderate relationship between digital literacy and mental well-being (r = .363), alongside a significant negative weak correlation between digital literacy and self-isolation, a phubbing sub-dimension (r =-.133). Regression analyses demonstrated that digital literacy significantly predicts both mental well-being (β =.363) and phubbing-related self-isolation (β = -.133). The findings imply that enhanced digital literacy may mitigate phubbing behaviors while simultaneously promoting mental well-being among young athletes. The implications for digital citizenship education and athlete development programs should be discussed.

## 1 Introduction

The pervasive integration of digital technologies-including computers, smartphones, and mobile devices-has fundamentally transformed contemporary social interaction patterns. These technologies now serve as primary conduits for communication, work, learning, and entertainment across diverse populations (1). The rapid acceleration of technological advancement, coupled with its profound influence on daily social life, has generated significant social (2, 3) and psychological implications (4–6) for individuals, thus the construct of digital literacy has gained prominence as a critical competency for navigating the modern digital landscape.

Digital literacy encompasses the multifaceted ability to effectively utilize digital technologies in our increasingly connected world (7). More specifically, it refers to individuals’ capacity to optimally employ digital devices for accessing, identifying, managing, creating, and communicating information (8). This competency extends beyond mere technical proficiency to include critical understanding of appropriate and conscious technology use (9), encompassing knowledge of when, why, and for what duration technological devices should be employed (10). Contemporary conceptualizations of digital literacy emphasize its role in evaluating and organizing digital activities that are embedded within daily life experiences (11), particularly as mobile technologies enable increasingly complex digital interactions (12). A defining characteristic of digital literacy is its inherent adaptability (13), which has resulted in the progressive expansion of its conceptual boundaries and practical applications.

The technological evolution and extensive digitalization of society have established digital competency as essential for full participation in modern civic and economic life (14). Recognition that digital literacy skills are fundamental for successful adaptation to digital societies has positioned these competencies as crucial 21st-century skills for individual development and societal contribution (15). Consequently, digital literacy has become a cornerstone of educational policy in numerous countries, recognized as both a fundamental attribute of engaged citizenship and a prerequisite for workforce competitiveness (16). This recognition has prompted many national governments to prioritize digital literacy acquisition in their policy frameworks, driven by both civic engagement and economic development imperatives (17).

Over recent years, on the other hand, the inappropriate utilization of digital technologies has given rise to problematic behaviors, most importantly “phubbing”- a portmanteau of “phone” and “snubbing” (18). Phubbing is characterized by individuals’ tendency to ignore their immediate social environment and avoid meaningful interpersonal communication by focusing attention on their smartphones during social interactions (19–21). Research alludes to the fact that Fear of Missing Out (FoMO) may serve as a psychological trigger for phubbing behaviors, compelling individuals to prioritize smartphone engagement over face-to-face social interaction (22). On the other hand, it has been highlighted that the inappropriate use of digital technologies and applications can lead to various psychosocial problems (23–27). Research findings point to their particularly concerning implications for mental health outcomes (28–32).

Mental well-being, as defined by the World Health Organization (33) represents a state of mental wellness that enables individuals to cope effectively with life stressors, realize their potential, engage in productive learning and work, and contribute meaningfully to their communities. This construct encompasses the capacity to utilize one’s skills for societal benefit, establish positive relationships, and maintain inner equilibrium alongside personal responsibility (34). Mental well-being fundamentally concerns an individual’s psychological state, including their emotional experiences, beliefs, motivations, and behavioral patterns (35). It reflects both the quality and nature of mental experiences individuals acquire throughout their lives (36). Research indicates that individuals with elevated mental well-being demonstrate superior positive psychological skills in self-relationships and interpersonal interactions, exhibiting greater effectiveness in stress management (37).

The sporting domain has not remained immune to digital and technological advancement, with these developments permeating all aspects of athletic life. Young university athletes naturally engage with technological tools and digital media to follow developments across social, sporting, and scientific domains. The literature mentions that digital literacy awareness enables more effective utilization of cognitive processes including productivity enhancement, sound decision-making, problem-solving, and solution-oriented thinking (38, 39). On top of that, digital literacy has been associated with numerous individual and social benefits (40–44).

Despite growing interest in digital literacy research, previous studies have predominantly employed summative assessment approaches, with limited attention to formative evaluation of students’ digital skill mastery (45). Emerging research indicates that phubbing behaviors may negatively impact individual development and impede the formation and maintenance of close relationships (46, 47). However, our understanding of mental well-being in the digital context remains limited (48). Given this research gap, we hypothesize that digital literacy awareness among young athlete-students may facilitate healthy social relationship formation and support optimal mental well-being levels, promoting healthier developmental processes in both athletic and social domains. This study, therefore, aimed to examine the relationships between digital literacy levels and both phubbing behaviors and mental well-being among university student-athletes. Given the scarcity of research simultaneously addressing digital literacy, phubbing, and mental well-being variables, the findings are expected to contribute meaningfully to the existing literature and inform future research directions. Based on the framework and empirical evidence reviewed above, the following hypotheses were formulated:

Hypothesis 1: Digital literacy significantly predicts mental well-being.
Hypothesis 2: Digital literacy significantly predicts nomophobia (a sub-dimension of phubbing).
Hypothesis 3: Digital literacy significantly predicts interpersonal conflict (a sub-dimension of phubbing).
Hypothesis 4: Digital literacy significantly predicts self-isolation (a sub-dimension of phubbing).
Hypothesis 5: Digital literacy significantly predicts problem awareness (a sub-dimension of phubbing).

## 2 Materials and methods

### 2.1 Data collection instruments

#### 2.1.1 Three validated measurement instruments were utilized to assess the study variables, each described below

##### 2.1.1.1 Digital literacy scale

Digital literacy was assessed through the scale originally developed by Ng (49) and subsequently adapted to Turkish by Ustundag et al. (50). This unidimensional instrument comprises 10 items rated on a 5-point Likert scale (1 = strongly disagree to 5 = strongly agree). Higher scores indicate greater digital literacy competence, and the Turkish language adaptation demonstrated satisfactory psychometric properties in previous research.

##### 2.1.1.2 Generic scale of phubbing

Phubbing behaviors were measured via the scale developed by Chotpitayasunondh and Douglas (51) and adapted to Turkish by Orhan-Goksun (52). This multidimensional instrument consists of 15 items rated on a 7-point Likert scale (1 = never to 7 = always) across four distinct sub-dimensions: nomophobia (fear of being without mobile phone contact), interpersonal conflict (conflicts arising from phone use), self-isolation (withdrawal from social interactions due to phone use), and problem awareness (recognition of problematic phone use patterns). Higher scores on each sub-dimension indicate greater levels of the respective phubbing behavior.

##### 2.1.1.3 Warwick-Edinburgh mental well-being scale short form

Mental well-being was assessed through the short form developed by Tennant et al. (53) and adapted to Turkish by Demirtas and Baytemir (34). This unidimensional scale comprises 7 items rated on a 5-point Likert scale (1 = none of the time to 5 = all of the time). The scale measures positive aspects of mental health, including positive feelings and functioning. Higher scores indicate superior mental well-being.

### 2.2 Research design

The study employed a correlational survey design, which belongs to the category of relational screening models aimed at determining the existence and degree of covariation between two or more variables (54). This non-experimental design was selected as appropriate for examining the relationships between digital literacy, phubbing behaviors, and mental well-being without manipulating the variables of interest.

### 2.3 Participants

The study sample consisted of 224 university students enrolled in sports sciences programs at a state university in Turkey. Participants ranged in age from 18 to 26 years (_M_ = 20.89, _SD_ = 1.90), with 109 females (48.7%) and 115 males (51.3%) represented. All participants were recruited through convenience sampling and provided informed consent prior to participation. Inclusion criteria required participants to be currently enrolled undergraduate students in sports sciences programs and to possess regular smartphone usage patterns.

### 2.4 Data collection procedure

Data collection was conducted during the 2024 academic year following institutional review board approval. Participants completed the survey instruments during regularly scheduled class periods, with data collection supervised by trained research assistants. The survey battery required approximately 30 minutes to complete. Participants were informed of their right to withdraw from the study at any time without penalty, and confidentiality was assured through anonymous data collection procedures.

### 2.5 Statistical analysis

Data analysis proceeded through several stages to ensure data quality and appropriate statistical modeling. Initially, missing data patterns were examined, and cases with excessive missing values (>10%) were excluded from analysis. Normality of the data distribution was evaluated through examination of skewness and kurtosis statistics. The psychometric properties of the scales were evaluated in greater detail through reliability analyses (Cronbach’s alpha coefficients) and confirmatory factor analysis (CFA) was conducted to validate the factor structure of each measurement instrument using maximum likelihood estimation. Statistical analyses included descriptive statistics (means, standard deviations, frequencies), Pearson product-moment correlations, and regression analyses. Mean scores were calculated for each scale and used in subsequent analyses. Regression analyses were conducted to test the study hypotheses, with digital literacy as the predictor variable and mental well-being and phubbing sub-dimensions as criterion variables. Statistical significance was set at p <.05.

## 3 Results

### 3.1 Measurement model validation

Confirmatory factor analysis (CFA) was conducted to evaluate the congruence between the hypothesized factor structure of each measurement instrument and the empirical data (55). The results demonstrated acceptable model fit across all three scales (**Table 1**).

**Table 1:** Confirmatory factor analysis results for scales.

Scales	CMIN/DF	CFI	GFI	IFI	AGFI	TLI	RMSEA
Digital Literacy (DLS)	2.084	.946	.948	.947	.904	.919	.070
Mental Well-being (WEMWBS-SF)	2.096	.966	.968	.967	.926	.940	.070
Phubbing (GSP)	2.029	.945	.915	.946	.873	.929	.068

For the Digital Literacy Scale, fit indices indicated adequate model fit: χ²/df = 2.084, CFI = .946, GFI = .948, IFI = .947, AGFI = .904, TLI = .919, and RMSEA = .070. The Warwick-Edinburgh Mental Well-being Scale similarly demonstrated satisfactory fit: χ²/df = 2.096, CFI = .966, GFI = .968, IFI = .967, AGFI = .926, TLI = .940, and RMSEA = .070. The Generic Scale of Phubbing exhibited acceptable fit indices: χ²/df = 2.029, CFI = .945, GFI = .915, IFI = .946, AGFI = .873, TLI = .929, and RMSEA = .068. These values satisfy established criteria for acceptable model fit (56, 57), with CFI and TLI values approaching or exceeding.95, RMSEA values below.08, and χ²/df ratios below 3.0 (58–61).

### 3.2 Data distribution and reliability assessment

Examination of data distribution characteristics showed that skewness and kurtosis values for all study variables fell within acceptable ranges (± 1.0), supporting the assumption of normal distribution necessary for parametric statistical analyses (62, 63). Internal consistency reliability, assessed through Cronbach’s alpha coefficients, demonstrated satisfactory reliability across all measures (**Table 2**). Reliability coefficients ranged from α = .724 to α = .858, all exceeding the minimum threshold of.70 recommended for research purposes (64).

**Table 2:** Skewness, Kurtosis, and Cronbach’s Alpha (α) Values for Scale Domains.

Variables	Skewness	Kurtosis	Cronbach’s alpha (α)
Digital Literacy	-.108	-.396	.819
Mental Well-being	-.460	-.168	.786
Nomophobia	-.128	-.879	.774
Interpersonal Conflict	.822	-.302	.828
Self-isolation	.455	-.602	.858
Problem Awareness	.243	-.803	.724

### 3.3 Descriptive statistics and correlational analysis

Pearson product-moment correlations were computed to examine the bivariate relationships among study variables (54). Correlation coefficients are typically interpreted as follows: 0 < r < 0.30 indicates a weak relationship, 0.30 < r < 0.70 a moderate relationship, and 0.70 < r < +1 a strong relationship (65).

As shown in **Table 3**, the correlational analysis unveiled several significant relationships. Digital literacy demonstrated a significant positive moderate correlation with mental well-being (r = .363, p <.01), indicating that individuals with higher digital literacy levels tend to report greater mental well-being. Additionally, a significant negative weak correlation emerged between digital literacy and self-isolation, a sub-dimension of phubbing (r = -.133, p <.05), meaning that higher digital literacy could be associated with reduced tendencies toward social isolation in digital contexts.

**Table 3:** Correlation analysis results of the research variables.

N = 224	1	2	3	4	5	6
Digital Literacy	–					
Mental Well-being	.363^**^	–				
Nomophobia	.117	.119	–			
Interpersonal Conflict	-.066	.012	.390^**^	–		
Self-isolation	-.133^*^	.015	.261^**^	.624^**^	–	
Problem Awareness	-.123	-.051	.386^**^	.632^**^	.534^**^	–

^**^ p<.01; ^*^ p<.05. 1: Digital Literacy, 2: Mental Well-being, 3: Nomophobia, 4: Interpersonal Conflict, 5: Self-isolation, 6: Problem Awareness.

### 3.4 Regression analysis results

A series of simple linear regression analyses were conducted to test the study hypotheses, examining the predictive utility of digital literacy for mental well-being and each phubbing sub-dimension (**Table 4**).

**Table 4:** Regression analysis results of the research variables.

Part 1								
**Model**	**B**	**Std.** **Error**	**Beta** **(β)**	**t**	**p**	**VIF**	p=.000^***^ F_(1-222)_= 33.726 D-W=1.842	R=.363 R^2^=.132 Adj. R^2^= .128
(Constant)	2.428	.251	–	9.681	.000	–		
Digital Literacy	.396	.068	.363	5.807	.000^***^	1.000		
	*Dependent Variable: Mental Well-being*							
Part 2								
(Constant)	3.780	.540	–	7.000	.000	–	p=.080 F_(1-222)_=3.083 D-W=1.685	R=.117 R^2^=.014 Adj. R^2^= .009
Digital Literacy	.258	.147	.117	1.756	.080	1.000		
	*Dependent Variable: Nomophobia*							
Part 3								
(Constant)	3.095	.543	–	5.702	.000	–	p=.328 F_(1-222)_=.959 D-W=1.748	R=.066 R^2^=.004 Adj. R^2^= .000
Digital Literacy	-.145	.148	-.066	-.979	.328	1.000		
	*Dependent Variable: Interpersonal Conflict*							
Part 4								
(Constant)	4.133	.575	**-**	7.188	.000	–	p=.047^*^ F_(1-222)_=4.002 D-W=2.043	R=.133 R^2^=.018 Adj. R^2^= .013
Digital Literacy	-.313	.156	-.133	-2.001	.047^*^	1.000		
	*Dependent Variable: Self-isolation*							
Part 5								
(Constant)	4.650	.588	–	7.902	.000	–	p=.066 F_(1-222)_= 3.414 D-W=1.828	R=.123 R^2^=.015 Adj. R^2^= .011
Digital Literacy	-.296	.160	-.123	-1.848	.066	1.000		
	*Dependent Variable: Problem Awareness*							

^***^p<.001; ^*^p<.05; Beta (β), Standardized coefficients; D-W, Durbin Watson; VIF, Variance Inflation Factor.

#### 3.4.1 Hypothesis 1: digital literacy predicting mental well-being

The regression model examining digital literacy as a predictor of mental well-being was statistically significant, (F_(df=1.222)_ = 33.726, p <.001). Digital literacy accounted for 13.2% of the variance in mental well-being scores (R² =.132),. The standardized regression coefficient indicated that digital literacy was a significant positive predictor (β = .363, t = 5.807, p=.000), supporting Hypothesis 1. This finding denotes that each unit increase in digital literacy is associated with a corresponding increase in mental well-being.

#### 3.4.2 Hypothesis 2: digital literacy predicting nomophobia

The regression analysis for nomophobia as the criterion variable did not achieve statistical significance (F_(df=1.222)_ = 3.083; p >.05). Digital literacy did not significantly predict nomophobia levels among participants, failing to support Hypothesis 2.

#### 3.4.3 Hypothesis 3: digital literacy predicting interpersonal conflict

The regression model with interpersonal conflict as the dependent variable was not statistically significant (F_(df=1.222)_ = .959; p >.05). Digital literacy did not emerge as a significant predictor of interpersonal conflict related to phubbing behaviors, so Hypothesis 3 was not supported.

#### 3.4.4 Hypothesis 4: digital literacy predicting self-isolation

The regression analysis revealed a statistically significant model (F_(df=1.222)_ =4.002; p = .047). Digital literacy explained approximately 2% of the variance in self-isolation scores (R² = .018). The standardized regression coefficient indicated that digital literacy was a significant negative predictor of self-isolation (β = -.133, t = -2.001, p = .047), supporting Hypothesis 4. This result suggests that higher levels of digital literacy are associated with reduced self-isolation behaviors.

#### 3.4.5 Hypothesis 5: digital literacy predicting problem awareness

The regression model examining problem awareness as the outcome variable approached but did not achieve statistical significance (F_(df=1.222)_ = 3.414; p >.05). Digital literacy did not significantly predict problem awareness levels, failing to support Hypothesis 5.

Overall, the regression analyses provided support for only two of the five study hypotheses. Digital literacy emerged as a significant positive predictor of mental well-being and a significant negative predictor of self-isolation, whereas it failed to predict nomophobia, interpersonal conflict, or problem awareness dimensions of phubbing behavior.

## 4 Discussion

This investigation examined the interrelationships among digital literacy, phubbing behaviors, and mental well-being within a sample of young university athletes. The findings contribute to our understanding of how digital competencies may influence both positive psychological outcomes and problematic technology-related behaviors in this unique population.

The primary finding confirmed a significant positive association between digital literacy and mental well-being, with digital literacy accounting for 13.2% of the variance in mental well-being scores. This relationship provides empirical support for Hypothesis 1 and aligns with emerging frameworks that position digital literacy as a protective factor in the digital age. Although direct comparative research within athlete populations remains rather limited, our findings are consistent with Meng et al. (66), who demonstrated comparable positive effects of digital literacy on mental well-being among preschool children, which could mean this relationship may be robust across developmental stages. The observed association between digital literacy and mental well-being can be understood through several mechanisms. Enhanced digital literacy may facilitate more purposeful and mindful technology engagement, reducing the cognitive load associated with navigating digital environments (67). Additionally, digitally literate individuals may possess superior skills for accessing mental health resources, maintaining social connections through digital platforms, and avoiding potentially harmful online content (68). This finding extends previous research demonstrating positive associations between digital literacy and related psychological constructs, including creativity and innovation (69), individual creative traits (70), psychological capital (71), communication competence (72), and self-efficacy beliefs (73). Besides, community-based digital literacy interventions have demonstrated efficacy in enhancing cognitive functioning (74), thus indicating that the benefits of digital competency extend beyond mere technical proficiency to encompass broader mental health outcomes. For university athletes, who must navigate both academic and athletic demands in the process of maintaining social relationships, enhanced digital literacy may provide crucial skills for managing multiple digital identities and communication channels effectively.

A second key finding was the significant negative association between digital literacy and self-isolation (a phubbing sub-dimension), with digital literacy negatively predicting this behavior, thus providing support for Hypothesis 4, which implies that digital literacy may serve as a protective factor against certain aspects of problematic smartphone use. This finding aligns with models proposing that digital literacy encompasses not merely technical skills but also critical awareness of technology’s social and psychological impacts (75). Digitally literate individuals may possess enhanced metacognitive awareness of their technology use patterns, enabling them to recognize when digital engagement becomes socially isolating and to employ self-regulatory strategies accordingly (76, 77). Because Today, digital literacy is an essential component of everyone’s personal and professional lives to survive and thrive in the digital world (78).

Looking at similar research in the literature, previous studies established links between various psychological factors and phubbing behaviors, including boredom and Fear of Missing Out (FoMO) (79), loneliness and smartphone addiction (80), and excessive internet or gaming engagement (22, 81–85). The observed negative relationship between digital literacy and self-isolation might arise from the broader benefits of digital literacy, such as improved critical thinking about technology use or enhanced online self-regulation, which have been reported in various contexts (67, 68, 75–77, 86–90). However, digital literacy did not significantly predict other phubbing dimensions like nomophobia, interpersonal conflict, or problem awareness in our sample, indicating a more nuanced role of digital literacy in mitigating specific facets of phubbing.

### 4.1 Implications for university athletes

The unique context of university athletes presents particular considerations for digital literacy and mental well-being relationships. Athletes face distinctive pressures related to performance, social media presence, and maintaining relationships across multiple social contexts (teammates, coaches, family, peers). Enhanced digital literacy may be especially valuable for this population in managing online reputation, accessing performance-related information, and maintaining social connections during travel and competition periods.

The finding that digital literacy predicts reduced self-isolation has particular relevance for athletes, who depend heavily on team cohesion and social support for optimal performance. Athletes who engage in self-isolating phubbing behaviors may compromise team dynamics and miss opportunities for crucial social learning and peer support.

### 4.2 Limitations and future directions

Several limitations warrant acknowledgment. The cross-sectional design precludes causal inferences about the relationships between digital literacy and outcome variables. Longitudinal research would provide stronger evidence for the protective effects of digital literacy over time. Additionally, the relatively modest effect sizes observed (particularly for the self-isolation relationship) evince that digital literacy represents one factor among many influencing these outcomes. The sample’s focus on university athletes limits generalizability to broader populations, though it provides valuable insights into this specific demographic. Future research should examine these relationships across diverse samples and consider potential moderating variables such as sport type, competitive level, and cultural context. The measurement of digital literacy through self-report instruments, though validated, may not capture the full complexity of digital competencies. Future studies might benefit from incorporating behavioral measures or performance-based assessments of digital skills. Finally, as the study was conducted with Turkish university students, cultural factors influencing digital technology attitudes and mental well-being perceptions may limit the direct applicability of findings to other cultural settings.

## 5 Conclusion

This study provides evidence that digital literacy serves as a significant predictor of both enhanced mental well-being and reduced self-isolation behaviors among young university athletes. The findings have important implications for educational practice and mental health promotion in higher education settings. Institutional investments in digital literacy education may yield dual benefits: improving students’ technological competencies whilst concurrently promoting psychological well-being and healthy social engagement. For universities, particularly athletic programs, integrating digital literacy initiatives that emphasize critical reflection on technology use, mindful engagement, and digital ethics-beyond mere technical skills—may foster healthier social behaviors and contribute to overall student development. As digital technologies become increasingly embedded in daily life, fostering these competencies is crucial for young adults’ psychosocial adjustment.

## Data Availability

The raw data supporting the conclusions of this article will be made available by the authors, without undue reservation.
